# Thirty-year trends and outcome of isolated versus combined group 2 pulmonary hypertension after cardiac transplantation

**DOI:** 10.3389/fcvm.2022.841025

**Published:** 2022-12-02

**Authors:** Amine Nasri, Jocelyn Dupuis, Michel Carrier, Normand Racine, Marie-Claude Parent, Anique Ducharme, Annik Fortier, Leslie Hausermann, Michel White, Maxime Tremblay-Gravel

**Affiliations:** ^1^Montreal Heart Institute Research Center, Montreal, QC, Canada; ^2^Department of Medicine, Faculty of Medicine, Université de Montréal, Montreal, QC, Canada; ^3^Department of Cardiac Surgery, Montreal Heart Institute, Université de Montréal, Montreal, QC, Canada; ^4^Montreal Health Innovations Coordinating Center, Montreal, QC, Canada

**Keywords:** pulmonary hypertension, cardiac transplant, heart failure, right heart catheterization, outcomes

## Abstract

**Aim:**

To investigate the effect of the new definition of pulmonary hypertension (PH) and new pulmonary vascular resistance (PVR) thresholds on the prevalence, clinical characteristics, and events following cardiac transplantation (CTx) over 30 years.

**Methods:**

Patients who underwent CTx between 1983 and 2014 for whom invasive hemodynamic data was available were analyzed (*n* = 342). Patients transplanted between 1983 and 1998 were classified as early era and those transplanted between 1999 and 2014 were classified as recent era. Group 2 PH was diagnosed in the presence of a mean pulmonary artery pressure (mPAP) > 20 mmHg and pulmonary capillary wedge pressure (PCWP) > 15 mmHg. Isolated post capillary PH (Ipc-PH) was defined as PVR ≤ 2 wood units and combined pre and post capillary PH (Cpc-PH) was defined PVR > 2 wood units. Moderate to severe PH was defined as mPAP ≥ 35 mmHg. The primary outcome was 30-day mortality and long-term mortality according to type and severity of PH. Proportions were analyzed using the chi-square test, and survival analyses were performed using Kaplan-Meier curves and compared using the logrank test.

**Results:**

The prevalence of PH in patients transplanted in the early era was 89.1%, whilst 84.2% of patients transplanted in the recent era had PH (*p* = 0.3914). There was no difference in the prevalence of a pre-capillary component according to era (*p* = 0.4001), but severe PH was more common in the early era (51.1% [early] vs 38.0% [recent] *p* = 0.0151). Thirty-day and long-term  mortality  were  not  significantly  associated  with severity or type of PH. There was a trend toward increased 30-day mortality in mild PH (10.1%), compared to no PH (4.4%) and moderate to severe PH (6.6%; *p* = 0.0653). Long-term mortality did not differ according to the severity of PH (*p* = 0.1480). There were no significant differences in 30-day or long-term mortality in IpcPH compared to CpcPH (*p* = 0.3974 vs *p* = 0.5767, respectively).

**Conclusion:**

Over 30 years, PH has remained very prevalent before CTx. The presence, severity, and type (pre- vs post-capillary) of PH is not significantly associated with short- or long-term mortality.

## Graphical Abstract

Prevalence and outcomes of group 2 PH in cardiac transplant recipients over 30 years. 
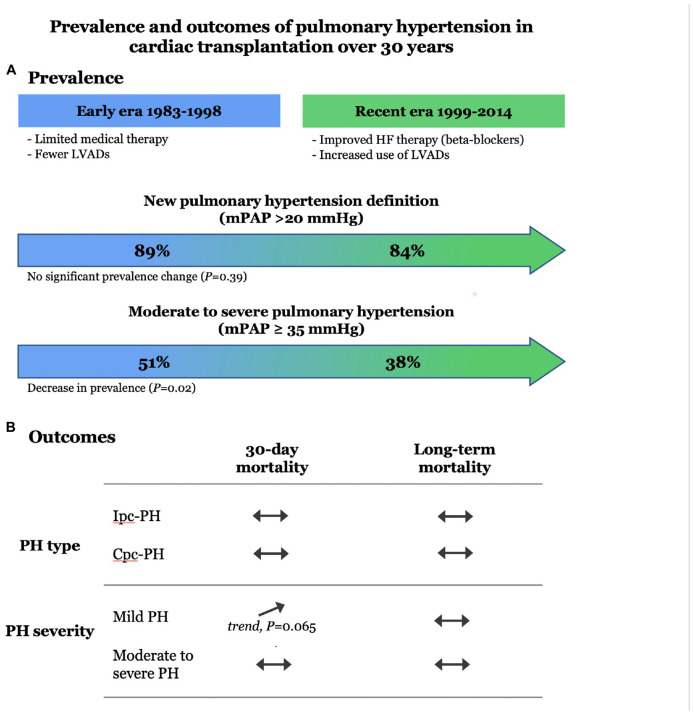

## Introduction

Cardiac transplantation (CTx) remains the preferred contemporary treatment for end-stage heart failure (HF) (1–3). The selection of CTx recipients and donors is influenced by the presence and the severity of group 2 pulmonary hypertension (PH) (4). The prevalence of group 2 PH has reached 75% in patients listed for cardiac transplantation (CTx) and the most recent ISHLT registry data reported a mean pulmonary vascular resistance (PVR) of 2.1 wood units (WU) preceding CTx (5).

Several studies have shown that even mild degree of pre-transplant PH may adversely influence survival after CTx, while it had no impact in others (6–18). Data from the United Network for Organ Sharing (UNOS) registry showed that a PVR ≥ 2.5 woods units (WU) was a significant risk factor for early mortality, but not for 10-year mortality (19). In contrast, some recent data from the International Society for Heart and Lung Transplantation (ISHLT) reported a small but a significant effect of a trans-pulmonary pressure gradient (TPG) > 12 mmHg on 10-year mortality (20). A recent retrospective analysis of the UNOS database reported a deleterious impact of “low” mean pulmonary artery pressure (mPAP) in the presence of high PVR on 30-day mortality after CTx (21). However, discrimination between isolated post capillary PH (Ipc-PH) and combined pre and post capillary PH (Cpc-PH), and the severity of PH, as well as the long-term impact of PH on events were not investigated in that report. Furthermore, there have been no studies investigating the impacts of pre-transplant PH on events and outcomes beyond 10 years following CTx.

In 2018, the World Society of Pulmonary Hypertension (WSPH) proposed to decrease the diagnostic threshold value for mPAP from ≥ 25 to > 20 mmHg (22). Furthermore, in 2022, the European Society of Cardiology (ESC) and European Respiratory Society (ERS) PH guidelines recommended to decrease the PVR threshold to discriminate between Ipc-PH and Cpc-PH from 3 WU to 2 WU, in order to reflect the lowest prognostically relevant threshold for PVR (23). As such, these recent recommendations suggested to diagnose Ipc-PH in the presence of an mPAP > 20 mmHg, a pulmonary capillary wedge pressure (PCWP) > 15 mmHg and PVR ≤ 2 WU while Cpc-PH was defined by a similar magnitude of mPAP and PCWP but in the presence of a PVR > 2 WU.

Despite the insightful data from the ISHLT registry, the impact of the new definition and sub-classifications of group 2 PH on the prevalence, clinical characteristics, and events in patients listed and transplanted since the availability of cyclosporine in 1983 have not been investigated. In addition, the effects of the presence and the magnitude of Ipc-PH and Cpc-PH on 30-day and long-term cardiovascular events and mortality have been incompletely studied. The primary objective of this study was to investigate the impacts of the new definition and sub-classifications of group 2 PH on the prevalence, clinical characteristics, and outcomes of patients studied prior CTx and followed over a period of 30 years in a single cardiac transplant center. The secondary objective was to assess the effects of the presence and severity of PH on major adverse cardiac events (MACE) and all-cause mortality.

## Materials and methods

This study was a retrospective, single center, non-interventional study. The population consisted of 342 adult (age ≥ 18 years) patients who received an orthotopic CTx at the Montreal Heart Institute between January 1983 and December 2014. All patients who received a first CTx and followed at the hospital were included in this analysis. Patients with incomplete information pre-transplantation and/or lost in follow-up were excluded from this report. This study was performed with the approval by the local ethics board at the Montreal Heart Institute.

Right heart catheterization (RHC) prior to CTx was predominantly performed in the catheterization laboratory via right femoral vein. If more than one catheterization was performed, we used the closest test prior to CTx for analysis. Hemodynamic data collected during RHC included systolic pulmonary artery pressure (sPAP), mPAP, diastolic pulmonary artery pressure (dPAP), PCWP, central venous pressure (CVP), TPG, diastolic pulmonary gradient (DPG), PVR, cardiac output CO) and heart rate (HR). The right ventricular stroke work index (RVSWI) was calculated by the following formula: (mPAP – mRAP) X SVI. Pulmonary artery pulsatily index (PAPI) was calculated using by the following formula: (sPAP-dPAP)/RAP. Pulmonary artery compliance was calculated using the following formula: (CO/HR)/(sPAP-dPAP).

Patients were classified as having PH based on their most recent RHC prior to CTx. Patients with a durable left ventricular assist device (LVAD) were also classified based on the last right heart catheterization completed prior to CTx. We classified patients according to the new definition of PH. Complete hemodynamic profile was performed and the selected parameters were computed. Patients with a mPAP ≤ 20 mmHg were classified as non-PH. Patients were classified with isolated post capillary PH (Ipc-PH) in the presence of a PCWP > 15 mmHg, mPAP > 20 mmHg, and a PVR ≤ 2 WU. Lastly, patients were diagnosed with Cpc-PH in the presence of PCWP > 15 mmHg, a mPAP > 20 mmHg, and a PVR > 2 WU. The prevalence of the new classification of group 2 PH was compared with patients classified using the old definition of PH (mPAP ≥ 25 mmHg) according to ISHLT guidelines. Moderate to severe PH was based on the distribution of PH in our study population and was defined by mPAP ≥ 35 mmHg.

Clinical events and all-cause mortality were computed for the whole period and according to the era of transplantation. Patients transplanted between 1983 and 1998 were included in the “early” era while those transplanted between 1999 and 2014 were classified as “recent” era. The secondary outcomes were MACE. For the purpose of this study, MACE was defined as allograft left ventricular dysfunction, stroke, transient ischemic attack, acute coronary syndrome, pulmonary embolism, and peripheral arterial embolism.

### Statistical analysis

Baseline characteristics were collected at time 0, defined as the date of the right heart catheterization indicating pre-transplant PH. Variables are presented as mean ± SD or frequencies and percentages and were compared using student *t*-tests, one way analysis of variance (ANOVA), or chi-square test, where appropriate. Kaplan–Meier curves for all-cause mortality were plotted according to PH classification and compared using the log-rank test. Two-tailed *p* values of <0.05 were considered statistically significant. Statistical analyses were performed using SAS version 9.4 software.

## Results

### Study population

The clinical characteristics of the study population are presented in Table 1. A total of 342 patients (early era, *n* = 184; recent era, *n* = 158) were followed for a mean duration of 9.5 years. The proportion of female recipients was lower in the early era. Subjects transplanted in the early era presented a higher rate of smoking but a lower prevalence of dyslipidemia. Patients transplanted in the recent era exhibited a higher rate of inotrope use and left ventricular assist device implantation prior to transplant. Patients transplanted in the recent era received a graft from older donors although there was no difference in the recipients’ age.

**TABLE 1:** Clinical characteristics of the study population.

	Early era (1983–1998)			Recent era (1999–2014)			
	No PH (*n* = 20)	Ipc-PH (*n* = 55)	Cpc-PH (*n* = 109)	No PH (*n* = 25)	Ipc-PH (*n* = 44)	Cpc-PH (*n* = 89)	*p*-value
**Demographics**							
Age at CTx (years)	45.9 ± 10.2	48.3 ± 9.7	46.8 ± 9.6	45.4 ± 15.9	49.5 ± 11.0	48.4 ± 13.1	0.5923
Male sex (*n*, %)	18 (90)	49 (89)	88 (81)	19 (76)	630 (68)	69 (78)	0.1309
Caucasian (*n*, %)	19 (95)	53 (96)	109 (100)	16 (89)	32 (100)	67 (94)	0.0590
**Clinical parameters (*n*, %)**							
Indication for HT, Ischemic (*n*, %)	14 (70)	31 (56)	59 (54)	5 (20)	18 (41)	38 (43)	0.0049
Diabetes mellitus (*n*, %)	3 (15)	5 (10)	11 (11)	0 (0)	6 (18)	15 (19)	0.4406
BMI, (kg/m^2^)	24.9 ± 4.2	23.7 ± 3.7	24.2 ± 3.8	24.5 ± 3.7	24.7 ± 4.3	25.3 ± 3.8	0.2161
Dyslipidemia	9 (45)	19 (35)	31 (28)	11 (44)	23 (53)	47 (53)	0.0053
Hypertension, (mmHg)	4 (20)	9 (16)	17 (16)	5 (20)	11 (26)	21 (24)	0.6304
History of smoking	14 (74)	40 (83)	63 (74)	10 (40)	20 (45)	36 (40)	<0.0001
**NYHA class pre-CTx (*n*, %)**							
1	0 (0)	0 (0)	1 (1)	0 (0)	1 (3)	1 (1)	0.6380
2	0 (0)	1 (2)	3 (3)	3 (13)	4 (10)	4 (5)	
3	14 (70)	36 (65)	66 (61)	13 (54)	25 (63)	49 (59)	
4	6 (30)	18 (33)	39 (36)	8 (33)	10 (25)	29 (35)	
**Pre-transplant laboratories**							
Glomerular filtration rate, (ml/min/1.73m^2^)	70.8 ± 29.8	65.4 ± 19.5	67.6 ± 20.5	77.7 ± 32.3	69.7 ± 28.6	71.0 ± 28.1	0.5210
LDL-cholesterol, (mmol/l)	2.9 ± 1.0	3.3 ± 1.1	3.1 ± 1.4	2.1 ± 0.8	2.2 ± 0.9	2.1 ± 0.9	<0.0001
Triglycerides, (mmol/l)	1.64 ± 0.81	1.77 ± 1.33	1.36 ± 0.77	1.23 ± 0.53	1.49 ± 1.12	1.29 ± 0.71	0.0946
**Transplant related parameters**							
Donor age, (years)	33.5 ± 9.9	30.7 ± 12.1	26.5 ± 10.5	35.7 ± 17.4	39.7 ± 14.8	38.0 ± 14.8	<0.0001
Ischemic time, (minutes)	137 ± 54	131 ± 45	143 ± 50	132 ± 61	141 ± 56	142 ± 53	0.7420
LVAD (*n*, %)	0 (0)	0 (0)	2 (2)	6 (24)	6 (14)	17 (19)	<0.0001
Pre-CTx IABP (*n*, %)	2 (10)	4 (7)	10 (9)	4 (16)	4 (9)	10 (11)	0.8843
Donor weight (kg)	73 ± 12	70 ± 11	69 ± 14	80 ± 20	73 ± 17	77 ± 16	0.0018
Donor weight/recipient ratio	0.99 ± 0.26	1.04 ± 0.29	1.00 ± 0.23	1.16 ± 0.27	1.08 ± 0.24	1.07 ± 0.25	0.0725
Pre-CTx inotropes (*n*, %)	6 (30)	33 (33)	22 (34)	13 (52)	41 (51)	22 (44)	0.0846
Female donor/male recipient (*n*, %)	3 (15)	17 (31)	22 (20)	6 (24)	8 (18)	18 (20)	0.5580
**Medication post-CTx (*n*, %)**							
Cyclosporine	20 (100)	49 (89)	102 (94)	8 (32)	20 (46)	41 (46)	<0.0001
Tacrolimus	0 (0)	0 (0)	1 (1)	10 (40)	7 (16)	21 (24)	<0.0001
MMF	3 (15)	5 (9)	10 (9)	16 (64)	28 (64)	56 (63)	<0.0001
Azathioprine	14 (70)	35 (64)	73 (67)	0 (0)	0 (0)	0 (0)	<0.0001
ACEi/ARB	7 (35)	10 (18)	33 (30)	3 (12)	3 (7)	10 (11)	0.0010
Calcium-channel blocker	0 (0)	0 (0)	0 (0)	0 (0)	4 (9)	1 (1)	0.0008
Loop diuretics	12 (60)	32 (58)	75 (69)	11 (44)	14 (32)	37 (42)	0.0001
Anticoagulants	0 (0)	6 (11)	5 (5)	7 (28)	7 (16)	10 (11)	0.0060
Anti-platelets	2 (10)	6 (11)	8 (7)	7 (28)	10 (23)	18 (20)	0.0193
Statins	0 (0)	1 (2)	1 (1)	11 (44)	12 (27)	44 (49)	<0.0001

BMI, body mass index; Cpc-PH, combined pre and post capillary PH; CTx, cardiac transplantation; DB, diabetes; HT, heart transplantation; IABP, intra-aortic balloon pump; Ipc-PH, Isolated post capillary PH; LVAD, left ventricular assist device; No PH, no pulmonary hypertension; NYHA, New York Heart Association. Values are mean ± SD or *n* (%). *p*-values represent the overall comparisons between the six groups by one-way ANOVA.

Right heart catheterization was performed a median of 96 days (lower quartile = 33; upper quartile = 185 days) prior to CTx in the early era and 84 days (lower quartile = 31; upper quartile = 164.5) days in the recent era (*p* = NS).

**FIGURE 1:**
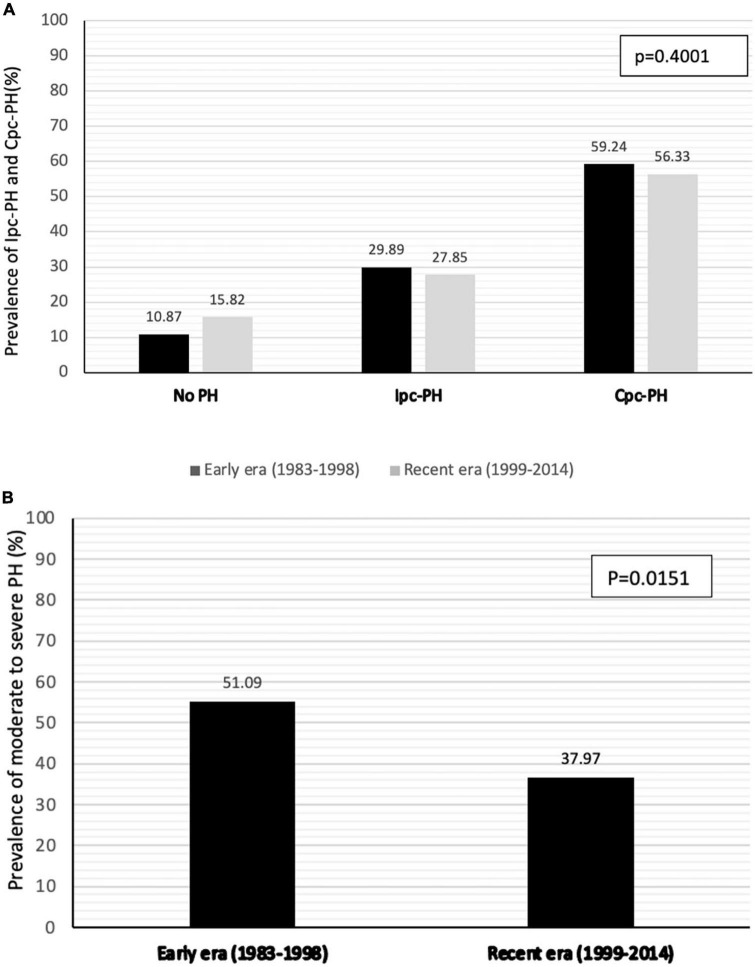
Prevalence of PH and pre-capillary component **(A)** and prevalence of moderate to severe PH (mPAP > 35mmHg) **(B)** according to era of transplantation.

The hemodynamic characteristics in relationship with the type and severity of PH for both early and recent eras are presented in Table 2. According to the new definition, the prevalence of PH was 89.1% in patients transplanted in the early era and 84.2% in the recent era (*p* = 0.3914) (Figure 1). When compared with the old definition, the use of a cut-off value of mPAP > 20mmHg (instead of ≥ 25 mmHg) in the new definition increased the prevalence of PH by 7.6% (old definition, 79.2%; new definition, 86.8%) in the whole cohort. In those with PH, the use of a cut-off value of 2 WU (instead of 3 WU) increased the prevalence of Cpc-PH by 27.6% (old definition, 39.1%; new definition, 66.6%) in the whole cohort. There was no significant difference in the prevalence of Ipc-PH vs Cpc-PH in early vs recent eras. While the prevalence of PH was similar in both eras, moderate to severe PH, defined by a mPAP ≥ 35 mmHg was 13.1% higher in patients transplanted in early era compared to the recent era (51.1% vs 38.0% *p* = 0.0151).

**TABLE 2:** Hemodynamic parameters in relationship with the type and the severity of pulmonary hypertension for the early and recent era.

	Early era (1983–1998)					Recent era (1999–2014)					
	No-PH (*n* = 20)	Ipc-PH mild (*n* = 38)	Cpc-PH Mild (*n* = 32)	Ipc-PH severe (*n* = 17)	Cpc-PH severe (*n* = 77)	No-PH (*n* = 25)	Ipc-PH mild (*n* = 36)	Cpc-PH Mild (*n* = 37)	Ipc-PH severe (*n* = 8)	Cpc-PH severe (*n* = 52)	*p*-value
HR (bpm)	87.0 ± 17.9	85.1 ± 13.2	82.2 ± 14.2	86.1 ± 14.2	87.0 ± 14.3	82.6 ± 10.2	79.6 ± 9.4	5.3 ± 9.0	82.5 ± 9.1	81.2 ± 11.0	0.0845
RAP (mmHg)	9.1 ± 4.9	11.3 ± 6.0	13.4 ± 7.5	14.8 ± 4.5	14.6 ± 7.1	8.80 ± 4.9	11.9 ± 7.0	16.0 ± 6.6	16.0 ± 8.2	20.0 ± 6.7	<0.0001
sPAP (mmHg)	29.9 ± 5.9	42.2 ± 7.5	45.3 ± 9.1	52.3 ± 11.8	61.8 ± 11.2	25.9 ± 5.8	41.8 ± 7.6	44.6 ± 10.3	66.3 ± 12.4	58.8 ± 10.7	<0.0001
dPAP (mmHg)	10.7 ± 4.2	18.8 ± 5.3	20.0 ± 5.6	24.8 ± 5.3	28.8 ± 5.3	9.80 ± 3.3	19.3 ± 4.2	20.1 ± 5.3	27.5 ± 2.2	28.5 ± 65.8	<0.0001
mPAP (mmHg)	17.1 ± 2.4	27.8 ± 4.0	29.2 ± 3.0	37.6 ± 3.2	42.6 ± 6.0	16.1 ± 3.0	28.0 ± 4.1	28.7 ± 3.6	44.3 ± 4.9	42.4 ± 6.3	<0.0001
CO (l/min)	4.22 ± 1.43	4.08 ± 1.14	3.32 ± 1.05	3.82 ± 1.46	3.26 ± 1.14	3.73 ± 1.36	3.99 ± 1.60	3.50 ± 1.29	3.99 ± 1.03	3.06 ± 1.18	<0.0001
PCWP (mmHg)	13.7 ± 5.2	23.3 ± 5.6	20.6 ± 6.4	26.8 ± 6.6	29.1 ± 6.6	11.4 ± 3.2	22.0 ± 5.4	19.6 ± 4.6	37.4 ± 4.4	28.0 ± 7.8	<0.0001
TPG (mmHg)	3.4 ± 5.1	4.5 ± 4.5	8.6 ± 6.5	10.8 ± 8.2	13.5 ± 5.7	4.8 ± 2.9	6.0 ± 3.9	9.1 ± 3.6	6.9 ± 4.0	14.3 ± 8.3	<0.0001
PVR (WU)	1.6 ± 0.8	1.3 ± 0.5	3.0 ± 0.8	1.6 ± 0.3	4.2 ± 2.12	1.7 ± 1.0	1.4 ± 0.4	3.2 ± 0.9	1.5 ± 0.4	4.3 ± 2.3	<0.0001
RAP/PWCP	0.75 ± 0.51	0.52 ± 0.31	0.71 ± 0.49	0.59 ± 0.24	0.52 ± 0.26	0.77 ± 0.36	0.58 ± 0.41	0.84 ± 0.41	0.44 ± 0.25	0.77 ± 0.38	0.0001
RVSWI (g*m/m^2^)	253 ± 155	428 ± 216	367 ± 227	557 ± 242	615 ± 412	220 ± 162	434 ± 228	287 ± 175	745 ± 354	450 ± 202	<0.0001
PAPI	3.2 ± 2.8	3.6 ± 4.5	3.1 ± 3.0	2.2 ± 1.4	3.2 ± 3.2	2.6 ± 2.5	3.1 ± 3.0	2.0 ± 1.6	4.3 ± 4.9	1.8 ± 1.3	0.0081
Pulmonary compliance	2.9 ± 1.4	2.2 ± 0.9	1.8 ± 0.9	2.2 ± 2.4	1.1 ± 1.0	4.1 ± 6.1	2.4 ± 1.1	1.9 ± 1.2	1.3 ± 0.3	1.5 ± 1.4	<0.0001

CO, cardiac output; Cpc-PH, combined post and precapillary pulmonary hypertension; RAP, right atrial pressure; dPAP, diastolic pulmonary artery pressure; DPG, diastolic pressure gradient; HR, heart rate; Ipc-PH, isolated post capillary pulmonary hypertension; mPAP, mean pulmonary artery pressure; No PH, no pulmonary hypertension; PCWP, pulmonary capillary wedge pressure; PVR, pulmonary vascular resistance; RVSWI, right ventricular stroke work index; sPAP, systolic pulmonary artery pressure; TPG, transpulmonary pressure gradient. *P* values represent the overall comparisons between the groups.

There were 31 patients who underwent LVAD implantation prior to heart transplant (29/31 in the recent era), of whom 6 had RHC data after LVAD implantation. The effects of the LVAD implantation on pulmonary pressure and vascular resistance, as well as specific adverse events, are presented in the Supplementary Table 1. There were no statistical difference in sPAP, mPAP or PVR between the 6 patients with hemodynamic data while on LVAD (*n* = 6) compared to the remainder of the cohort (*n* = 336). When compared with hemodynamics obtained prior to device implantation, we observed that LVAD lowered mPAP in all patients and lowered PVR in 5/6 patients. Four patients (75%) presented adverse events between 17 and 237 days following CTx.

### Mortality and major adverse cardiac events

**FIGURE 2:**
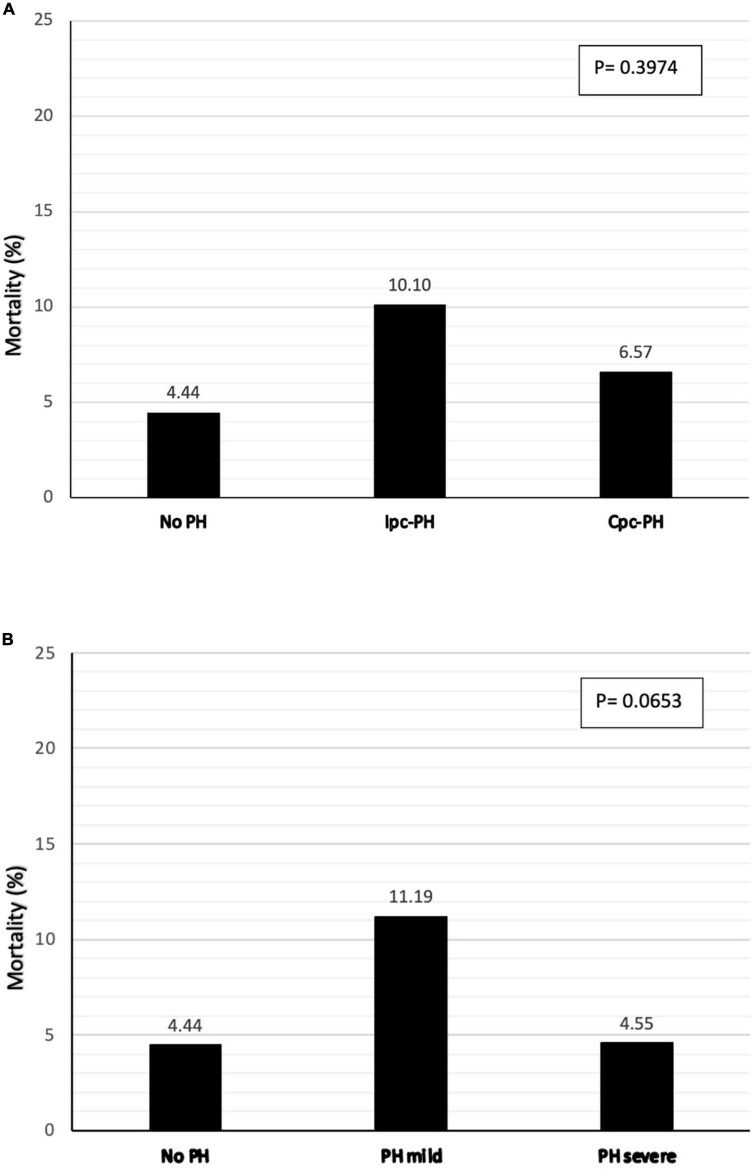
Thirty-day mortality following CTx according to the type **(A)** and severity of PH **(B)**.

Short term mortality (30-day) was not significantly impacted by the severity of PH or the presence of a pre-capillary component (Figure 2). Only a trend toward higher 30-day mortality was found in patients with mild pH compared to moderate-severe or absence of PH (*p* = 0.0653).

**FIGURE 3:**
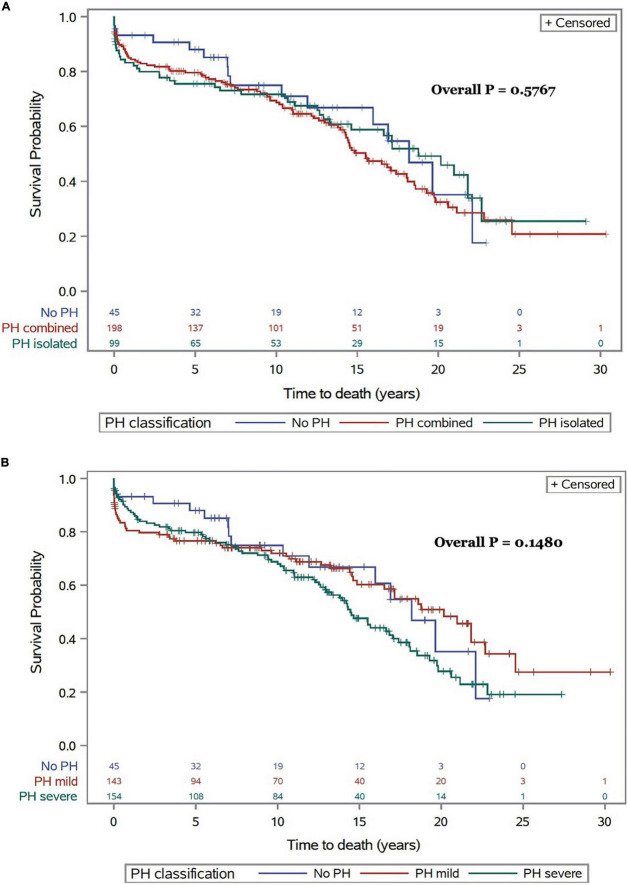
All-cause mortality following CTx for the whole cohort and for the entire follow-up period in relationship with type **(A)** and severity of PH **(B)**.

Survival estimates for patients with and without PH and in relationship with the severity and type of PH for the whole study period are presented in Figure 3. There were no significant survival differences in patients with Cpc-PH compared to Ipc-PH and no PH (0.5767). Similarly, no significant differences in mortality were observed with regards to the presence or absence of mild or severe PH over 30 years (*p* = 0.1480).

The impact of the type of PH on adverse events at 1- and 3-years are presented in Table 3. There was no significant association between PH and stroke, myocardial infarction, peripheral emboli, pulmonary embolism, renal failure or retransplantation. There was a trend toward a higher rate of graft dysfunction at 3-year in the early era, especially in patients with PH (*p* = 0.0539). Similarly, treated acute cellular rejection (ACR) episodes were more common in the early era regardless of PH (*p* < 0.0001).

**TABLE 3:** Selected events up to 3 years following CTx according to the era of transplantation and the severity of PH.

	Early era (1983–1998)			Recent era (1999–2014)			
	No PH (*n* = 20)	Ipc-PH (*n* = 55)	Cpc-PH (*n* = 109)	No PH (*n* = 25)	Ipc-PH (*n* = 44)	Cpc-PH (*n* = 89)	*p*-value
**LVEF < 50%**							
At 1 year	2 (10)	8 (15)	14 (13)	3 (12)	2 (5)	6 (7)	0.4486
At 3 years	3 (15)	10 (18)	24 (22)	3 (12)	3 (7)	7 (8)	0.0539
**GFR < 60**							
At 1 year	11 (55)	34 (62)	59 (54)	12 (48)	23 (52)	44 (49)	0.7786
At 3 years	14 (70)	38 (69)	71 (65)	12 (48)	25 (57)	46 (52)	0.1459
**Retransplantation**							
At 1 year	0 (0)	0 (0)	1 (1)	0 (0)	0 (0)	0 (0)	0.8289
At 3 years	0 (0)	0 (0)	1 (1)	0 (0)	0 (0)	0 (0)	0.8289
**Treated ACR**							
At 1 year	6 (30)	24 (44)	44 (40)	4 (16)	3 (7)	13 (15)	<0.0001
At 3 years	6 (30)	25 (45)	46 (42)	4 (16)	5 (11)	15 (17)	<0.0001

Cpc-PH, combined post and precapillary pulmonary hypertension; ACR, acute cellular rejection; GFR, glomerular filtration rate; Ipc-PH, isolated post capillary pulmonary hypertension; LVEF, left ventricular ejection fraction; No PH, no pulmonary hypertension.

## Discussion

This study completed in a single transplant center with a follow-up beyond 30 years reported on the clinical and hemodynamic characteristics, and outcomes associated with the presence or absence of PH, its sub-type, and its severity following CTx. These analyses were performed according to the new PH definition (2018) and pre-capillary classification (2022). The use of the new PH definition increased the overall prevalence of PH by an absolute of 7.1%. Among subjects with PH, the new PVR cut-off increased the proportion of Cpc-PH by 27.6%. Although the prevalence of Ipc-PH or Cpc-PH did not change significantly across eras, the prevalence of moderate to severe PH, defined here as mPAP ≥ 35 mmHg, whether isolated or combined, was reduced in the recent era. There was no significant impact of the presence of PH pre-CTx on 30-day and long-term mortality following CTx.

The presence of group 2 PH, or PH due to left heart diseases, is very common in patients with various HF phenotypes (4, 24). Data from the UNOS reported a mean PVR of 2.1 in patients listed for CTx (19). This value has not significantly changed over the last decades. The presence and magnitude of PH, assessed by either mean PAP and/or PVR have been associated with some adverse outcomes in patients with HF and following CTx (19, 21, 25, 26). Similarly, data from the Health Care Registry have published a significant increase in CV events when PVR reach 2.2 WU or higher. In 2018, the definitions task force of the WSPH suggested to reduce mean PAP cut-off value to > 20 mmHg instead of ≥ 25 mmHg for the diagnosis of PH (22). This new cut-off value has been pragmatically based on the normal distribution of mPAP values and on the fact that many studies have reported some worse outcomes in patients with mPAP > 20 mmHg (27). Similarly, worse outcomes were reported with PVR as low as 2 WU, justifying this lower threshold for the diagnosis of Cpc-PH (23).

In this investigation, we reported on the clinical characteristics, hemodynamic data and on outcomes in patients with isolated and combined group 2 PH based on the new definitions. Patients with Cpc-PH presented with more advanced disease both hemodynamically and clinically, including more advanced RV dysfunction as previously reported. In this investigation we report significant changes in the clinical parameters in patients with Cpc-PH compared with those with Ipc-PH. Indeed, patients with Cpc-PH yielded a higher CVP/wedge ratio, lower PAPi and lower RVSWi suggesting more advanced RV failure.

Here, we report a nearly 15% decrease in the rate of moderate to severe PH in the recent era. This observation is likely related with better heart failure management including an increased use of mineralocorticoid antagonists, more frequent use of PDE5 inhibitors such as sildenafil and tadalafil, and the use of LVAD as bridge to transplantation.

The presence of moderate to severe PH did not confer an adverse prognosis, possibly related to the U-shaped distribution of PA pressures, where patients with more advanced heart failure exhibit decreased cardiac output generating low pressures despite increased resistance. Nevertheless, Cpc-PH, reflective of pulmonary vascular resistance and less dependent on cardiac output, did not yield any significant associations with outcomes.

In this study, we reported on selected clinical events according to the type and severity of PH in the early versus recent era. No significant differences in the event rate post-CTx were observed in relationship with the presence of either Ipc-PH versus Cpc-PH pre-CTx. Nevertheless, patients transplanted in the early era exhibited a trend toward an increased rate of graft dysfunction defined by an LVEF < 50% at 3 years. The reasons for this remain unclear but are most likely multifactorial. Parameters such as better pharmacologic management, early detection of antibody-mediated rejection and more efficient immunosuppressive strategies in the high-risk patients may have contributed to this finding.

The observations reported here somewhat diverge from the recent publication from Crawford et al. (27). In the latter study, the investigators analyzed more than 32 thousand patients included in the UNOS database. The authors reported no significant impact of a low mPAP (< 25 mmHg), but a significant impact of higher mPAP (≥ 25 mmHg) on 30 days and 1-year mortality rate. Also, patients with a mPAP < 25 mmHg but with a PVR ≥ 3 WU had a significant increase in 30-days mortality rate, but no difference in 1-year mortality. Despite a large sample size, detailed hemodynamic characteristic and events were not reported beyond 1 year following CTx in that later study (27). Here we report that regardless of the subtype of group 2 PH, there is no association with short or long term outcomes. As such, our findings provide some important additional information on the impact of various phenotypes of PH, and some further insights on events collected long-term following CTx. Finally, our observations provide novel data on the long-term temporal changes of group 2 PH preceding and following CTx.

### Limitations

This study was a retrospective investigation and as such some significant limitations need to be outlined. Despite data computed in a single center since 1983, the sample size was rather small and most likely underpowered to conclude on clinical events and mortality. Nevertheless, this analysis provides the longest follow-up available on the impact of PH diagnosed prior to CTx and its relationship with events following CTx. The clinical and hemodynamic data were acquired at different time points prior to CTx which may have increased the variability of our observations. Patients transplanted in the recent era had an infrequent yet a greater use of LVAD compared with patients transplant in the early era. However, only six patients had repeated RHC prior to CTx. The magnitude of decrease in pulmonary pressure observed in these patients is unlikely to have significantly interfere with our findings. The pre-CTx hemodynamic data were collected for clinical purpose and as such were not recorded in systematic fashion. This and the fact the study was performed by many different operators may have contributed to some significant variability in the hemodynamic data. Analyses were not adjusted for potential confounding variables, such as changes in immunosuppressive medications over time. Finally, duration of heart failure, which contributes to pulmonary arteriolar remodeling and increased resistance, was not collected in our data base and as such is not reported here.

## Conclusion

The prevalence of both Ipc-PC and Cpc-PH is very common preceding CTx. Although the proportion of patients with severe PH has significantly decreased in the recent era, the overall prevalence of PH has not changed over 30 years. The presence, severity, and type (pre- vs post-capillary) of PH is not significantly associated with short- or long-term mortality. Novel strategies to mitigate PH pre-CTx and better post CTx management are needed to decrease morbidity and mortality in these high-risk patients.

## Data availability statement

The original contributions presented in the study are included in the article/Supplementary material, further inquiries can be directed to the corresponding author.

## Ethics statement

This study was reviewed and approved by the Montreal Heart Institute Research Ethics and New Technology Development Committee.

## Author contributions

AN, JD, AD, MW, and MT-G designed the study. AN, MC, and AF collected the data. AN and AF did the formal analysis. MC and MW acquired the funding. NR, M-CP, AD, MW, and MT-G did the investigation. JD, AD, AF, MT-G, and MW worked on the methodology. LH and MW administrated the project. LH acquired the resources. JD, MC, AD, MW, and MT-G supervised the project. AN, NR, M-CP, MW, and MT-G validated. AN wrote the original draft. JD, NR, M-CP, AD, AF, MT-G, and MW did the review the writing and editing. All authors contributed to the article and approved the submitted version.

## Abbreviations

ANOVA: analysis of variance
CO: cardiac output
Cpc-PH: combined pre and post capillary PH
CTx: cardiac transplantation
CVP: central venous pressure
dPAP: diastolic pulmonary artery pressure
DPG: diastolic pulmonary gradient
HR: heart rate
Ipc-PH: isolated post capillary PH
ISHLT: International Society for Heart and Lung Transplantation
LVAD: left ventricular assist device
MACE: major adverse cardiac events
mPAP: mean pulmonary artery pressure
PCWP: pulmonary capillary wedge pressure
PH: pulmonary hypertension
PVR: pulmonary vascular resistance
RHC: right heart catheterisation
RVSWI: right ventricular stroke work index
sPAP: systolic pulmonary artery pressure
TPG: trans-pulmonary pressure gradient
UNOS: United Network for Organ Sharing
WSPH: World Society of Pulmonary Hypertension
WU: woods units.
