# Supplementary material for: Thirty-year trends and outcome of isolated versus combined group 2 pulmonary hypertension after cardiac transplantation
Source: Front Cardiovasc Med. 2022 Dec 2;9:841025. doi: 10.3389/fcvm.2022.841025 (PMC9755656; doi:10.3389/fcvm.2022.841025)
Supplement: Supplementary file 1 [file Table_1.DOCX]

**Supplemental Table 1. Hemodynamic data from LVAD patients before cardiac transplantation**

|  | **sPAP (RHC 1)** | **sPAP**  **(RHC 2)** | **mPAP (RHC 1)** | **mPAP (RHC 2)** | **PVR (RHC 1)** | **PVR (RHC 2)** | **Days to RHC** | **Days on LVAD** | **Events** |
| --- | --- | --- | --- | --- | --- | --- | --- | --- | --- |
| *Patient 1* | 40 | 30 | 33 | 19 | 3.1 | 2 | 89 | 124 | Pulmonary embolism 54 days after transplantation |
| *Patient 2* | 49 | 38 | 24 | 17 | 3.2 | 1.9 | 70 | 91 | None |
| *Patient 3* | 29 | 27 | 24 | 22 | 2.3 | 2 | 40 | 70 | Myocardial infarction 237 days after heart transplantation |
| *Patient 4* | 48 | 49 | 24 | 22 | 2.3 | 2 | 127 | 176 | Stroke 17 days after transplantation |
| *Patient 5* | 45 | 40 | 34 | 20 | 2 | 2 | 90 | 105 | None |
| *Patient 6* | 65 | 45 | 25 | 14 | 4.1 | 3.8 | 59 | 72 | Septic choc causing death 6 months after transplantation |

RHC 1 was done prior to LVAD implantation.

RHC 2 was done after LVAD implantation. This hemodynamic data was used for the analyses

Days to RHC: Total days on VAD before RHC.

Days on LVAD: Total days on VAD before receiving cardiac transplantation.

RAP, right atrial pressure; sPAP, systolic pulmonary artery pressure; mPAP, mean pulmonary artery pressure; PCWP, pulmonary capillary wedge pressure; PVR, pulmonary vascular resistance; RAP/PCWP, right atrial pressure / pulmonary capillary wedge pressure ratio; LVAD, left ventricular assist device; RHC, right heart catheterization.
