# Supplementary material for: Thirty-year trends and outcome of isolated versus combined group 2 pulmonary hypertension after cardiac transplantation
Source: Front Cardiovasc Med. 2022 Dec 2;9:841025. doi: 10.3389/fcvm.2022.841025 (PMC9755656; doi:10.3389/fcvm.2022.841025)
Supplement: Supplementary file 2 [file Table_2.DOCX]

**Supplemental Table 2. Selected events up to 3 years following CTx according to the era of transplantation and the severity of PH, not reported in Table 3**

|  | **Early era (1983-1998)** | | | **Recent era (1999- 2014)** | | |  |
| --- | --- | --- | --- | --- | --- | --- | --- |
|  | No PH (*n*=20) | Ipc-PH (*n*=99) | Cpc-PH (*n*=65) | No PH (*n*=25) | Ipc-PH (*n*=82) | Cpc-PH (*n*=51) | *p*-value |
| **Stroke** |  |  |  |  |  |  |  |
| At 1 year (*n*, %) | 0 (0) | 2 (4) | 1 (1) | 0 (0) | 0 (0) | 1 (1) | 0.5480 |
| At 3 years (*n*, %) | 0 (0) | 2 (4) | 2 (2) | 0 (0) | 0 (0) | 1 (1) | 0.6536 |
| **Myocardial infarction** |  |  |  |  |  |  |  |
| At 1 year (*n*, %) | 0 (0) | 0 (0) | 0 (0) | 0 (0) | 0 (0) | 0 (0) |  |
| At 3 years (*n*, %) | 0 (0) | 0 (0) | 0 (0) | 0 (0) | 0 (0) | 0 (0) |  |
| **Pulmonary embolism** |  |  |  |  |  |  |  |
| At 1 year (*n*, %) | 0 (0) | 0 (0) | 2 (2) | 1 (4) | 0 (0) | 0 (0) | 0.3268 |
| At 3 years (*n*, %) | 0 (0) | 0 (0) | 3 (3) | 1 (4) | 0 (0) | 0 (0) | 0.2558 |
| **Peripheral embolism** |  |  |  |  |  |  |  |
| At 1 year | 1 (5) | 2 (4) | 2 (2) | 0 (0) | 0 (0) | 2 (2) | 0.6927 |
| At 3 years | 2 (10) | 2 (4) | 4 (4) | 0 (0) | 0 (0) | 3 (3) | 0.3736 |

Cpc-PH, combined post and precapillary pulmonary hypertension; ACR, acute cellular rejection; GFR, glomerular filtration rate; Ipc-PH, isolated post capillary pulmonary hypertension; LVEF, left ventricular ejection fraction; No PH, no pulmonary hypertension.
